# Supplementary material for: ‘We are all serving the same Ugandans’: A nationwide mixed-methods evaluation of private sector surgical capacity in Uganda
Source: PLoS One. 2019 Oct 24;14(10):e0224215. doi: 10.1371/journal.pone.0224215 (PMC6812829; doi:10.1371/journal.pone.0224215)
Supplement: S1 Appendix — (PDF) [file pone.0224215.s001.pdf]

National Surgical, Obstetric and Anaesthesia Planning (NSOAP)
Semi-structured Hospital Interview Tool

Interviewee: **CEO/Hospital Director**
Name and title of interview subject:
Contact information:

Framing statement: The purpose of this interview is to understand the key factors affecting the provision of safe, affordable and timely surgical care – from a management perspective – at this facility. We would like to understand the challenges you face as well as the main areas for improvement at this facility?

INFRASTRUCTURE

1. **Describe your facility's infrastructure and how it affects your facility's ability to provide surgical care.**

   Prompts:
   - *How frequently does your facility experience interruptions in basic utilities (e.g running water, electricity)? How do you work around these interruptions?*
   - *What shortages in terms of essential supplies or surgical equipment does your facility face? (eg. XR, CT, basic labs). What accounts for these shortages?*
   - *Describe what processes are in place for equipment maintenance.*

2. **What are the key challenges your facility faces in terms of infrastructure?**

   Prompts:
   - *Does your facility face shortages in terms of space utilization (e.g. number of ORs, number of beds)?*

WORKFORCE

3. **Describe your facility's human resources and how they affect your ability to provide surgical care.**

   Prompts:
   - *What shortages, if any, does your facility face in terms of workforce (e.g surgeons, nurses, techs)?*
     - *What accounts for this workforce shortage?*
     - *What would your facility need (i.e. what specific healthcare providers) to more adequately address the surgical burden of disease?*
     - *What problems, if any, does your facility face in recruiting and retaining workforce? What attraction and retention strategies are in place?*
   - *How are non-surgeons/obstetricians used to assist with surgical care of patients?*

4. **Describe the working environment of your facility.**

   Prompts:
   - *How do you foster a positive, productive working environment?*
   - *How would you describe the quality of management/leadership within your facility?*
   - *Describe how governing bodies affect management of this facility? Both from hospital leadership and from Ministry of Health.*

5. **What are they key challenges your facility faces in terms of workforce?**

SERVICE DELIVERY

6. **Describe how your facility addresses or can improve quality of surgical care.**

   Prompts:
   - *What forms of quality control are in place at your facility? (e.g. infection control, checklists, Morbidity and Mortality report) What is the process for this? Who participates?*
   - *Describe how your facility incorporates feedback from patients? (e.g. patient satisfaction surveys)?*

7. **In what ways does your facility coordinate care with other partners (e.g NGOs, social work)?**
8. **What concerns for quality of care at this facility do you have?**

FINANCING

9. **What issues with affordability do your patients face in accessing surgical care**?

   Prompts:
   - *What parts of surgical care and services, if any, are patients required to pay for out of pocket?*
   - *What is the range of these costs? How often do patients pay these?*

10. **What is the process of budgeting for surgical care at your facility?**

    Prompts:
    - *How is the budget for surgical services determined?*
    - *How much of your annual budget is allocated to surgery?*
    - *What mechanisms are in place for re-evaluating the budget?*
    - *Who provides input into/participates in budgeting decisions? Do clinical providers have any stake in resource allocation?*

11. **What are the key challenges your facility faces in terms of financing?**

INFORMATION MANAGEMENT

12. **Describe the ways in which health information management systems affect your facility's ability to provide surgical care**

    Prompts:
    - *Describe the medical record keeping system at your facility.*
      - *Are charts readily accessible across multiple visits for the same patient?*
    - *What surgical data (clinical processes, cost, outcomes) are you required to report to the Ministry of Health?*
    - *How would you characterize the quality of data collection and management*

13. **What are the key challenges your facility faces in terms of information management?**

National Surgical, Obstetric and Anaesthesia Planning (NSOAP) Semi-structured Hospital Interview Tool

Interviewee: **Surgeon/Ob-Gyn**
Name and title of interview subject:
Contact information:

Framing statement: The purpose of this interview is to understand the key factors affecting the surgeon's ability to provide safe, affordable and timely surgical care at this facility. We would like to understand the challenges you face as well as the main areas for improvement here.

INFRASTRUCTURE

1. **Describe the process by which a patient comes to get care at this facility.**

   Prompts:
   - *How quickly, on average, are patients able to reach the facility?*
   - *By what mode of transport do patients typically arrive (e.g. ambulance, community, etc)?*
   - *How are patients referred into hospital? (e.g. ED, from smaller hospitals, from health posts)*

2. **Describe the process for transferring surgical patients to a higher-level facility.**

   Prompts:
   - *Why do patients have to be referred away to other facilities?*

3. **Describe your facility's infrastructure and how it affects your ability to provide surgical care.**

   Prompts:
   - *What factors affect your ability to make a diagnosis?*
   - *What factors affect your ability to carry out necessary procedures?*
   - *What factors affect your ability to provide adequate post-operative care?*
   - *What are innovations or work-arounds you use to circumvent these challenges?*
   - *Suggestions on what is meant by infrastructure:*
     - *Materials such as suture, staplers, implants*
     - *Equipment such as electrocautery, sterilizers, CT scanners*
     - *Space such as operating rooms, minor procedure rooms*
     - *Utilities such as oxygen, electricity*

4. **What are the key challenges you face in terms of surgical infrastructure?**

WORKFORCE

5. **Describe your facility's human resources and how they affect your ability to provide surgical care.**

   Prompts:
   - *How is the clinical work shared amongst the surgeons at this facility?*
   - *How are non-surgeons used to assist with surgical care of patients?*

6. **Describe the working environment of your facility.**

   Prompts:
   - *How do you foster a positive, productive working environment?*

- *How does this facility address clinician burnout?*
- *How would you describe the quality of management/leadership in your facility?*
- *Describe the career and administrative support you receive at this facility?*
- *Describe how governing bodies affect management of this facility? Both from hospital leadership and from Ministry of Health*

7. **What are they key challenges your facility faces in terms of workforce?**

SERVICE DELIVERY

8. **Describe how your facility addresses or can improve quality of care.**

Prompts:

- *What safety mechanisms do you employ in surgical care?*
- *What forms of quality control are in place at your facility? (e.g. infection control, checklists, Morbidity and Mortality report)*
- *Describe how your facility incorporates feedback from patients? (e.g. patient satisfaction surveys)?*

9. **What key challenges do your facility face in delivering safe, high quality surgical care?**

FINANCING

10. **How do costs of care affect your patients**?

Prompts:

- *What surgical services are patients required to pay for?*
  - *What pre-hospital costs do patients incur?*
  - *What in-hospital costs are patients required to pay out of pocket? (e.g. fees, materials, medications, diagnostic services, informal payments)*
  - *After discharge, what costs are patients required to pay out of pocket?*
- *What alternatives exist if the patient is or becomes unable to pay for services?*

11. **What are your views on the budget allocation for surgical care?**

Prompts:

- *How are you able to influence budgeting for the provision of care?*

12. **What are key challenges your facility faces in terms of financing surgery?**

INFORMATION MANAGEMENT

13. **What are key challenges you face in terms of accessing patient information?**

Prompts:

- *Do you have easy access to medical records related to a patient's current visit? Related to any past visits?*
- *How readily are charts accessible across multiple visits for the same patient?*

14. **What are your views on the information management system at your facility?**
15. **What are key challenges you face in terms of carrying out research?**

National Surgical, Obstetric and Anaesthesia Planning (NSOAP) Semi-structured Hospital Interview Tool

Interviewee: **Anaesthesia Provider**
Name and title of interview subject:
Contact information:

Framing statement: The purpose of this interview is to understand the key factors affecting the anaesthetists' ability to provide safe, affordable and timely anaesthesia care at this facility. We would like to understand the challenges you face as well as the main areas for improvement at this facility?

INFRASTRUCTURE

1. **Describe your facility's infrastructure and how it affects your facility's ability to provide anaesthesia care.**

   Prompts:
   - *What factors affect your ability to make a diagnosis?*
   - *What factors affect your ability to carry out necessary procedures?*
   - *What factors affect your ability to provide adequate post-operative care?*
     Suggestions on what is meant by infrastructure:
   - *Materials - endotracheal tubes, bougie*
   - *Equipment - bronchoscope, pulse oximeter, monitors, anaesthesia machine*
   - *Space - post-operative care unit, induction space, operating rooms*
   - *Utilities - oxygen, electricity*

2. **Describe your facility's infrastructure and how it affects your facility's ability to care for the critically ill patient?**

   Prompts:
   - *What advanced care services are available?*
   - *What mechanisms exist for the safe transfer of patients to a higher level of care?*
   - *What are problems you face with the safe and efficient transfer of critically ill patients to a higher level of care? What can be improved upon?*

3. **What are the key challenges you face in terms of anaesthesia infrastructure?**

WORKFORCE

4. **Describe your facility's human resources and how they affect your ability to provide anaesthesia care.**

   Prompts:
   - *How is the clinical work shared amongst the anaesthesia providers at this facility?*
   - *What happens when no anaesthesia is available?*
   - *How is the role of non-physician providers in anaesthesia care?*

5. **What is the capacity for this facility to provide anaesthesia training?**

   Prompts:
   - *How does/would such training affect the work at the hospital?*
   - *Who receives training in anaesthesia care at this facility? Medical students, general doctors, nurses, technicians?*

6. **Describe your working environment.**

Prompts:
- *How do you foster a positive, productive working environment?*
- *How does this facility address clinician burnout?*
- *Describe how governing bodies affect management of this facility? Both from hospital leadership and from Ministry of Health*

7. **What are the key challenges you face in terms of anaesthesia workforce?**

SERVICE DELIVERY

8. **Describe how your facility addresses or can improve quality of care.**

Prompts:
- *What safety mechanisms do you employ in anaesthesia care?*
- *What forms of quality control are in place at your facility? (e.g. infection control, checklists, Morbidity and Mortality report)*
- *How are health care providers held accountable for overall performance and results?*

9. **What key challenges do your facility face in delivering safe, high quality anaesthesia care?**

FINANCING

10. **How do costs of care affect your patients**?

Prompts:
- *What anaesthesia services are patients required to pay for?*
    - *What in-hospital costs are patients required to pay out of pocket? (e.g. fees, materials, medications, diagnostic services, informal payments)*
    - *After discharge, what costs are patients required to pay out of pocket?*
- *What alternatives exist if the patient is or becomes unable to pay for services?*

11. **What are your views on the budget allocation for anaesthesia care?**

Prompts:
- *How are you able to influence budgeting for the provision of care?*

12. **What are key challenges your facility faces in terms of financing anaesthetic care?**

INFORMATION MANAGEMENT

13. **What are key challenges you face in terms of accessing patient information?**

Prompts:
- *Do you have easy access to medical records related to a patient's current visit? Related to any past visits?*
- *How readily are charts accessible across multiple visits for the same patient?*

14. **What are your views on the information management system at your facility?**

National Surgical, Obstetric and Anaesthesia Planning (NSOAP) Semi-structured Hospital Interview Tool

Interviewee: **Surgical Nurse (Ward or Emergency Rom)**
Name and title of interview subject:
Contact information:

Framing statement: The purpose of this interview is to understand the key factors affecting the nurses' ability to provide safe, affordable and timely care for surgical patients at this facility. We would like to understand the challenges you face as well as the main areas for improvement at this facility?

INFRASTRUCTURE

1. **Describe the process by which a patient comes to get care at this facility.**

   Prompts:
   - *How quickly, on average, are patients able to reach the facility?*
   - *By what mode of transport do patients typically arrive (e.g. ambulance, community, etc)?*
   - *How are patients referred into hospital? (e.g. ED, from smaller hospitals, from health posts)*

2. **Describe the process for transferring surgical patients to a higher-level facility.**

   Prompts:
   - *Why do patients have to be referred away to other facilities?*
   - *What are the transfer criteria?*
   - *How often do referred patients successfully make it to the next facility?*
     - *What are the bottlenecks in the process?*

3. **Describe your facility's infrastructure and how it affects your ability to provide nursing care for surgical patients.**

   Prompts:
   - *What factors affect your ability to make a diagnosis?*
   - *What factors affect your ability to carry out necessary procedures?*
   - *What factors affect your ability to provide adequate post-operative care?*
   - *What are innovations or work-arounds you use to circumvent these challenges?*
   - *Suggestions on what is meant by infrastructure:*
     - *Materials such as medication, catheters*
     - *Equipment such as EKG*
     - *Space such as procedure rooms*
     - *Utilities such as oxygen, electricity*

4. **What are the key challenges you face in terms of surgical infrastructure?**

WORKFORCE

5. **Describe your facility's human resources and how they affect your ability to take care of surgical patients.**

   Prompts:
   - *What are the nurse-patient ratios?*
   - *Are there workforce shortages?*
   - *Do the skill/training level of nurses match surgical needs at this facility?*

6. **Describe the working environment of your facility.**

   Prompts:
   - *How do you foster a positive, productive working environment?*
   - *How would you describe the quality of management/leadership in your facility?*

7. **What are they key challenges your facility faces in terms of workforce?**

SERVICE DELIVERY

8. **In what ways do nurses at your facility participate in the delivery of pre- to post-operative surgical and anaesthesia care?**

   Prompts:
   - *How are patient care responsibilities divided between the wards, ICU, emergency ward, PACU, and outpatient department(s)?*
   - *What is the average nurse to patient ratio in each of these settings*

9. **Describe how your facility addresses or can improve quality of care.**
10. **What key challenges do your facility face in delivering safe, high quality surgical care?**

FINANCING

11. **How do costs of care affect your patients**?

    Prompts:
    - *What surgical services are patients required to pay for?*
      - *What pre-hospital costs do patients incur?*
      - *What in-hospital costs are patients required to pay out of pocket? (e.g. fees, materials, medications, diagnostic services, informal payments)*
      - *After discharge, what costs are patients required to pay out of pocket?*
    - *What alternatives exist if the patient is or becomes unable to pay for services?*

12. **What are your views on the budget allocation for surgical care?**

    Prompts:
    - *How are you able to influence budgeting for the provision of care?*

13. **What are key challenges your facility faces in terms of financing surgery?**

INFORMATION MANAGEMENT

14. **What are key challenges you face in terms of accessing patient information?**

    Prompts:
    - *Do you have easy access to medical records related to a patient's current visit? Related to any past visits?*
    - *How do you document intraoperative or post-operative clinical events?*
    - *Are you able to adequately review this documentation when needed?*
    - *How readily are charts accessible across multiple visits for the same patient?*

15. **What are your views on the information management system at your facility?**
