# Supplementary material for: ‘We are all serving the same Ugandans’: A nationwide mixed-methods evaluation of private sector surgical capacity in Uganda
Source: PLoS One. 2019 Oct 24;14(10):e0224215. doi: 10.1371/journal.pone.0224215 (PMC6812829; doi:10.1371/journal.pone.0224215)
Supplement: Attachment — Submitted filename: Response to Reviewers.docx [file pone.0224215.s002.docx]

September 22, 2019

To Whom It May Concern:

The authors thank the reviewers for this detailed and substantive review. We have taken into account the suggestions of the reviewers and have addressed the comments in turn in our italicized comments below.

We hope these edits have resulted in a stronger and clearer paper, and one that will contribute to the international dialogue on surgery and the public health imperative of surgical capacity building.

Furthermore, a complete and revised version of the manuscript is uploaded as is a document with track-changes allowing the reviewers to see in detail the adjustments and modifications that have been made.

Sincerely,

The authors

When submitting your revision, we need you to address these additional requirements.

Please ensure that your manuscript meets PLOS ONE's style requirements, including those for file naming. The PLOS ONE style templates can be found at

<http://www.journals.plos.org/plosone/s/file?id=wjVg/PLOSOne_formatting_sample_main_body.pdf> and <http://www.journals.plos.org/plosone/s/file?id=ba62/PLOSOne_formatting_sample_title_authors_affiliations.pdf>

*The manuscript has been updated and now meets PLOS ONE’s style requirements*

1. Please amend your current ethics statement to address the following concerns: Please explain why was written consent was not obtained, how you recorded/documented participant consent, and if the ethics committees/IRBs approved this consent procedure.

*The ethics statement has been updated and now reads as follows: “This study was deemed exempt from review by the Institutional Review Board (IRB) at Boston Children’s Hospital and approved by the IRBs at Mbarara University of Science and Technology and the Uganda National Council for Science and Technology. Additional approval was obtained from the Ministry of Health and Uganda Catholic Medical Bureau, where appropriate. Verbal informed consent was obtained from study participants. Due to the quality improvement and internal review nature of the process, as well as to preserve anonymity of participants, written consent was not obtained. Furthermore, all data were immediately de-identified on the individual and site-specific level to preserve anonymity.”*

2. Please amend your list of authors on the manuscript to ensure that each author is linked to an affiliation. Authors’ affiliations should reflect the institution where the work was done (if authors moved subsequently, you can also list the new affiliation stating “current affiliation:….” as necessary).

*Each author is linked to an affiliation and the corresponding author has been indicated with an **

Reviewer #1: This paper provides a capacity assessment for surgical care at private Ugandan hospitals, supplemented by qualitative data from key informants to give greater context to the challenges facilities face in service delivery. It addresses a need to understand the private hospital system that is often neglected or very difficult to obtain information from in similar settings. The paper is clear and well-structured with only some points needing clarification, but could do more to move the conversation forward towards actionable solutions and/or outlining next steps on the agenda.

*Thank you*

Major comments:

1. On sampling: Would be additionally helpful to understand the total distribution of public and private/PNFP hospitals across Uganda and understand what proportion of private facilities have been surveyed here. May also be relevant to have some understanding of who operates the private hospitals in the country – is it two or three major private entities or is facility operated by completely different entities? Understanding these aspects may lend greater credence to representativeness of the sample.

*Information regarding the total number of private and PNFP facilities is now included. It is difficult to generalize as to the ownership of the private/PNFP facilities in the country, however a sentence has also been added regarding the religious affiliation and governance of many of the PNFP facilities.*

2. In the implementation of the quantitative tool, were each of the items/processes/individuals in question observed or were individuals interviewed to answer these questions? This is implied, but it would be helpful to directly state this is indeed the case.

*The following sentence has been added to the methods section to clarify.* *“Information was collected through a combination of direct observation during hospital walk-throughs, as well as through in-person interviews.”*

3. Were group interviews managed or structured differently from individual interviews? For example, was it moderated in such a way as to ensure participation from all in the group? Or in other ways? What consideration was there of potential group effects (e.g. hierarchical structures among participants of a mixed group)? Or were group interviews conducted by cadre?

*This is an excellent point. A sentence has been added to indicate that group interviews were moderated such as to facilitate input from all participants. Unfortunately, however, it is not possible to completely mitigate the bias that group effect might have introduced.*

4. For qualitative interviews, it would be helpful to see a breakdown of the type of respondent by type of hospital (i.e. large/small, Private/PNFP, Bellwether/non-Bellwether facility, other relevant categories) being cautious not to allow for the identification of respondents. If only certain cadres responded for a particular type of facility, it may be a limitation of having only a single perspective for such cases.

*A breakdown of the interviews by private/PNFP and facility size is now provided in the methods section.*

5. Where are measures on ‘access to care’ as reported in the results collected across the quantitative and qualitative tools? It is unclear from where this information has been drawn given the tools’ focuses on infrastructure, service delivery, workforce, information management, and financing.

*The surgical assessment tool assesses “access to care” as a component of infrastructure – asking questions regarding catchment area, percentage of patient population that can reach the hospital within two hours. The qualitative tool also includes questions structured around the concept of access to care.*

6. On supply chain, is there information on what proportion of supplies are obtained via Joint Medical Stores, donations, and the open market? This could point to how much donations are relied upon, for example, and what issues to address to improve the supply chain (e.g. funding, pricing, or other agreements with stores, etc.).

*This is an important thing to acknowledge. Unfortunately, this information is not available. JMS is a monopoly and is certainly an area ripe for improvement.*

7. Across the paper, it would be helpful to distinguish when you are referring to clinical providers or the organization as provider, if that is the case. At some points, it is unclear whether answers came from clinical providers or hospital administrators (i.e, hospital directors, finance officers, etc.), though they would have distinctly different views and experiences on topics, such as staff training, contracts, service delivery, and costs.

*In the context of this manuscript, the term “providers” refers exclusively to clinical providers and not to the organization/facility or to administrators. Where hospitals or administrators are referred to, this is otherwise specified.*

8. Across the paper, it would be helpful in several cases to specify the numbers and/or percentages instead of stating “many hospitals” or “most hospitals”. This would provide clearer information more directly.

*Numbers and percentages have been added to clarify, particularly in the infrastructure section of the paper – though this information is also depicted in the figures.*

9. The barriers presented here are common in resource-constrained settings, and the authors have sufficiently linked their findings to other literature on the Ugandan health system. For at least some of these issues, the discussion could go on to discuss potential solutions that have been proposed or attempted in similar settings to move their agenda forward. There is some discussion comparing the public and private sectors – is there potential for solutions across public and private sectors given the larger understanding of the broader surgical care system that the information presented in the paper provides?

*The intent of this project was to better understand the capacity of the Ugandan private and PNFP sector to deliver safe, timely, and affordable surgical care. By highlighting the existing capacity of these sectors, we hope that this data contributes meaningfully to process improvement – exactly as you suggest. One sentence has been inserted to acknowledge that partially because of this project, coordination of care between sectors has been identified as a strategic priority for MOH. SOA workforce scale-up is also highlighted as an urgent need.*

Minor comments:

1. Clarification requested on data collection, line 87: What was the sampling frame from which these private/PNFP hospitals were selected? Are we to assume that the MOH has a comprehensive list of all private hospitals in the country which was used to select the sample? This may not be the case in some resource-constrained settings.

*This has been clarified. There is a master list of all hospitals in the country (including public, private, and PNFP faciltiaties)*

2. Line 176: Should be a comma, not a period after ‘ward’.

*Fixed. Thanks.*

3. To provide greater understanding of an individual’s perspective, could provide some description of the respondent’s cadre when providing quotes from qualitative interviews.

*We left this level of detail out to safeguard the anonymity of respondents.*

4. Line 241: It would be more helpful to identify how many facilities of those surveyed had set up this ‘stand-by’ group and expand on what proportion of providers in this group are split between public and private service delivery.

*The wording here has been amended to reflect that all facilities have an external bank of providers with whom they partner. Access statistics for SOA providers in the private and PNFP sector have also been inserted.*

5. The results of t-tests run for comparisons are not presented or described in the text? For which comparisons were these statistical tests conducted? And what were the findings?

*No p-values or tests of statistical significance are presented deliberately.*

6. Figure 1: Could some indication of population density be included in the map?

*The figure is designed in this fashion to mirror the figure presented in the public sector manuscripts. The authors feel that adding population density makes the figure slightly too busy and less interpretable.*

Other general comments / questions:

1. Is the distribution of public and private/PNFP hospitals across Uganda proportional to care utilization patterns? For example, if about half of Ugandans access care in the private sector, do private facilities also make up half of the country’s facilities or is something else driving disproportionate utilization? If there are fewer private facilities, but half of utilization still happens at these facilities, it may provide yet greater motivation for understanding the capacity of these facilities to provide quality care with consideration of demand.

*In strict numerical terms, there were 59 public hospitals and 90 private/PNFP hospitals; however, many of these private/PNFP sector facilities are disproportionally small so it is difficult to extrapolate this breakdown/distribution to countrywide care utilization.*

2. Were there differences in barriers or trends in capacity by region or type of hospital?

*There were no significant differences when the hospitals were broken down by type or region, though the significance of these conclusions is limited by power and thus why it is not presented.*

Reviewer #2: This article by Albutt, et al, provides a comprehensive assessment of capacity that includes evaluation in the context of bellwethers, operative log assessment, and structured interview. The overall goal is working in collaboration with the MOH to improve care, and the collaborative nature of the work is to be applauded. However, the manuscript lacks focus in the results section. It is unclear whether results are frim the quantitative or qualitative portion of the study and hence, reads more like a public health report than a scientific manuscript. I would recommend to the authors to completely re-write/re-present the results in an organized and systematic fashion with as much data presented as possible rather than summary results. This study has great value, but it needs some work in more deliberate presentation of results.

*Thank you for your feedback. We have attempted to address your concerns in the results by adding more numerical results data where possible and appropriate. Additional quantitative data is also depicted in the attached figures and embedded tables. Due to the mixed-methods nature of this research, the authors feel strongly that presenting the quantitative and qualitative data side by side enriches the study and gives context to current state of surgical capacity in Uganda. However, we are limited by the amount of information that can be provided and digested in the results in a succinct fashion. Furthermore, the quantitative and qualitative components of this study were conducted side by side and oftentimes overlapped thus many of the numerical quantitative results are presented with relevant qualitative context. For example, statements such as: “Hospital catchment populations were described as large (as many as several million people), far-reaching, and occasionally inclusive of refugee camps and neighboring countries” capture data elucidated from both the quantitative and qualitative portions of the assessment.*

*The strictly qualitative data is indicated by the presence of quotation marks.*

Some additional comments are listed below.

Abstract conclusion should read “As in Uganda’s public sector”, not “As Uganda’s public sector”

*Corrected*

Line 73-74 run-on sentence

*Corrected*

Line 75-77 reference that 29% of facilities in prior studies were PNFP but lists a huge number of references. Is this a meta-analysis or a result from one study? As much as I appreciate the extensive literature review of the authors, referencing 9-38 references is nonspecific.

*Prior to conducting the private/PNFP sector segment of this study, we conducted an extensive literature review of existing capacity assessments to see what percentage of them included private or PNFP facilities. There are many capacity assessments that strictly evaluate the government/public sector without mention of the private/PNFP sector which may represent upwards of half of care provision in the country. The statistic presented, 29.8% is the number of facilities in the available surgical assessments in sub-Saharan Africa that were private or PNFP.*

Line 85 Is there a reference to the prior study? If not, then the methods need to b fully described.

*The prior study is referenced – references 5 & 6*

Line 89 Is the stratified random sampling method utilized by the authors an established metholdology (i.e., provide a reference for the methodology).

*Stratified random sampling is an established form of probability-based sampling. Here is a reference for the methodology: stratified purposive sampling for mixed methods research (*[*https://www.ncbi.nlm.nih.gov/pmc/articles/PMC4012002/*](https://www.ncbi.nlm.nih.gov/pmc/articles/PMC4012002/)*)*

Line 90-93 How were the hospitals randomly sampled? Please describe the sampling methodology in detail. Were they randomly sampled or a convenience sample using criteria?

*See above.*

Line 129 “Interview transcriptions were cross-checked for accuracy and completeness.” By whom? How were they checked?
*This has been clarified*

While I found the study overall to be very comprehensive and collaborative, I found that the results were nondescript. The study uses qualitative and quantitative methodology, and it was unclear which was used for the presentation of results.

*We have attempted to address your concerns in the results by adding more numerical results data where possible and appropriate. Additional quantitative data is also depicted in the attached figures and embedded tables. Due to the mixed-methods nature of this research, the authors feel strongly that presenting the quantitative and qualitative data side by side enriches the study and gives context to current state of surgical capacity in Uganda. Furthermore, the quantitative and qualitative components of this study were conducted side by side and oftentimes overlapped. We feel that presenting the numerical quantitative results with relevant qualitative context adds value. The authors would argue, furthermore, that the interpretations of the data (from the quantitative assessment, qualitative assessment, or both) are equally important notwithstanding their quantitative or qualitative origin.*

It is unclear in the results what is taken from the quantitative survery and what is evaluated from the qualitiative assessment. For example, “often” under access—is this quantitative or qualititative? Both are described in the methodology. The catchment areas described, is this a known number (which it should be?) or an estimate form the qualitative assessment?

*The catchment populations for private and PNFP facilities are amorphous and do not follow any geographic boundaries and thus cannot be described in a precise number. Statements such as: “Hospital catchment populations were described as large (as many as several million people), far-reaching, and occasionally inclusive of refugee camps and neighboring countries” capture data elucidated from both the quantitative and qualitative portions of the assessment.*

Line 171 (as an example) Please provide quantitative results in table format.

*The bed capacity is detailed in Table 1 – hospital metrics*

Line 182 “Most facilities” – please be more definitive. This “most” is repeated throughout the results rather than definitive numbers.

*We have attempted to add numerical results throughout the results section in lieu of using the words most or many. Additional quantitative data is also depicted in the attached figures and embedded tables which are perhaps easier to understand than it being written out in the text. The remaining instances of “most” are almost always in direct reference to an accompanying figure.*

Despite the first portion of the study being a quantitative assessment of capacity, including resources, and procedures, the results section utilizes nondescript “most” rather than numerical results. It is unclear throughout the results section which results are qualitative and which are quantitative. As such, the vision of the background and methods is diluted.

*See above*

Line 259-261 If C-sections are 48% and laparotomies and open fracture reduction are only 4.9% and 2/0%, respectively, what are the other procedures being performed? Are they “major” or “minor” surgery? Does this reflect anything. then, on the selection of these procedures as bellwethers?

*A table has been created detailing the operative breakdown of cases. The breakdown of additional cases is included here. The inclusion of these three things as bellwethers has less to do with their percentage contribution to case volume and more to do with them serving as a proxy for a surgically capable system. Bellwether procedures are defined in the manuscript as: “A Bellwether procedure was defined as cesarean delivery, laparotomy, or open fracture treatment; procedures that serve as a proxy for surgical systems that are functioning at a level of complexity advanced enough to do most other surgical procedures”*

Line 262 “This is particularly common for orthopedic procedures: patients are
263 often temporized and referred, asked to wait for an orthopedic specialist to be called in from
264 another hospital, or are treated by a non-specialist.” Again, it is unclear if this is reported in the qualititate portion fo the study or if this is a conclusion drawn from the study, in which case, it should be in the discussion.

*This is data from the qualitative portion of the study, hence why it is presented in the results instead of the discussion. We have added in “providers note that” to better clarify.*

266-268 Please quantify this better, ideally in table format.

*A table has been created detailing the operative breakdown of cases.*

The discussion is well-written and a good summary.

*Thank you*

What does this study add to the literature? The discussion should focus more on how this study is different and how it adds to what is already known in the literature.

*Historically, little attention has been paid to understanding and improving the capacity of the private and PNFP sector which must be a critical component of ongoing capacity building efforts going forward. This manuscript details the capacity of the private and PNFP sector, which has never been done, and lends voices to those on the frontlines.*
